# Covid-19 vaccines in Italian public opinion: Identifying key issues using Twitter and Natural Language Processing

**DOI:** 10.1371/journal.pone.0277394

**Published:** 2022-11-17

**Authors:** Luisa Stracqualursi, Patrizia Agati

**Affiliations:** Department of Statistics, University of Bologna, Bologna, BO, Italy; Tokyo Institute of Technology: Tokyo Kogyo Daigaku, JAPAN

## Abstract

The COVID-19 pandemic has changed society and people’s lives. The vaccination campaign started December 27^*th*^ 2020 in Italy, together with most countries in the European Union. Social media platforms can offer relevant information about how citizens have experienced and perceived the availability of vaccines and the start of the vaccination campaign. This study aims to use machine learning methods to extract sentiments and topics relating to COVID-19 vaccination from Twitter. Between February and May 2021, we collected over 71,000 tweets containing vaccines-related keywords from Italian Twitter users. To get the dominant sentiment throughout the Italian population, spatial and temporal sentiment analysis was performed using VADER, highlighting sentiment fluctuations strongly influenced by news of vaccines’ side effects. Additionally, we investigated the opinions of Italians with respect to different vaccine brands. As a result, ‘Oxford-AstraZeneca’ vaccine was the least appreciated among people. The application of the Dynamic Latent Dirichlet Allocation (DLDA) model revealed three fundamental topics, which remained stable over time: vaccination plan info, usefulness of vaccinating and concerns about vaccines (risks, side effects and safety). To the best of our current knowledge, this one the first study on Twitter to identify opinions about COVID-19 vaccination in Italy and their progression over the first months of the vaccination campaign. Our results can help policymakers and research communities track public attitudes towards COVID-19 vaccines and help them make decisions to promote the vaccination campaign.

## 1 Introduction

SARS-CoV-2 infections are estimated to be already over 394 million worldwide, counting at least 5,7 million COVID-19 related deaths as of the February 1st 2022 [1]. Mortality aside, the disease has shown to be an unprecedented burden to both economies and healthcare systems in Italy [2]. The vaccination campaigns represented a new hope to put an end to the pandemic.

The Italian vaccination campaign started December 27^*th*^ 2020. Vaccines were firstly inoculated to healthcare workers and nursing home guests. Starting February 2021, the entire population was progressively involved following medical and social priorities [3].

In a coordinated effort with the EU Commission, the Italian government purchased—gradually and depending on availability—different brands of vaccines: Pfizer-BioNTech, Moderna, Oxford-AstraZeneca and Johnson&Johnson.

Despite all of the above were authorised both medically and politically by the European and Italian institutions, a number of people was not comfortable with the idea of being vaccinated and a minority of them refused the administration of the serum. This unleashed a harsh debate which inevitably landed on social media.

Thanks to almost 3.8 billion users across the globe [4], social media platforms are a precious data source and researchers have often profitably analysed comments extracted from e.g. Facebook [5], Twitter [6, 7], Instagram [8], etc. regarding political, business and healthcare issues.

This paper applies Natural Language Processing (NLP) techniques to explore major topics and sentiments of tweets about COVID-19 vaccines among Twitter users in Italy.

In this paper we aim to answer three main questions related to the first 4 months of the vaccination campaign in Italy:

- What has been the dominant sentiment towards COVID-19 vaccines? We responded through a sentiment analysis on vaccine-related Italian tweets, also detailing the sentiment in the various Italian regions. We used VADER as a sentiment analysis tool [9].
- Which brands of COVID-19 vaccine have been most talked about in Italy? Do people prefer any brands to others? In this regard, we explored the sentiment of Italian Twitter users towards different COVID-19 vaccine brands. We extracted vaccine brand hashtags from tweets and used VADER to analyse people’s preferences with respect to different brands.
- What were the main topics of discussion regarding COVID-19 vaccines? Was there any change in public opinion during the months of the vaccination campaign? With respect to this problem, we have used the Dynamic version of the Latent Dirichlet Allocation model (DLDA) [10], exploring the most popular themes and their evolution over time.

## 2 Methodology

### 2.1 The data

The aim of this paper is to investigate public opinion and perception on COVID-19 vaccines in Italy using tweets containing both #covid and #vaccine hashtags over the period 1^*st*^ February to 31^*st*^ May 2021.

Raw data was provided by ‘TrackMyHashtag’ [11] and a total of 73,688 tweets were collected. Each gathered tweet was written in Italian and, in addition to text content, included information about the date on which the tweet was created, the Twitter screen name of the user, the device used to post it, the number of re-tweets (‘reposted tweets’), the URLs of the Tweet, the follower number, and the likes number. Approximately 18,000 of the tweets collected also contained users’ tweeting location.

The Twitter dataset was processed and analysed with Python 3 following these steps:

1. *Raw data pre-processing*. Natural Language Processing (NLP) techniques were used to pre-process raw tweets, with the aim of cleaning the text and removing irrelevant information
2. *Sentiment Analysis*. With the aim to identify and extract attitudes, opinions, evaluations and emotions within the gathered tweets, VADER (Valence Aware Dictionary and sEntiment Reasoner) [9] was used to run Sentiment Analysis of tweets.
3. *Topic Extraction*. After proper pre-processing, Dynamic Latent Dirichlet Allocation (LDA) was applied to detect the main topics in the collection of tweets and their evolution over months.

### 2.2 Data pre-processing

A Tweet is a microblog message posted on Twitter. The text content of a Tweet has a limit to 280 characters, which is reduced to 257 characters if a link is included. Raw Tweets are highly unstructured and embed redundant information.

With the aim of converting raw data into an easily readable format that is to be used in Sentiment and Topic analyses, we applied the following pre-processing steps using NLP techniques with the aid of Python:

1. Removed mentions, URLs, email addresses and hashtags
2. Replaced HTML characters with Unicode equivalent (such as replacing ‘&amp;’ with ‘&’)
3. Removed HTML tags (such as < *div* >, < *p* >, etc.)
4. Removed unnecessary line breaks
5. Removed special characters and punctuation except for exclamation points (the exclamation point is the only punctuation marks to which the used VADER lexicon is sensitive)
6. Removed words that are numbers
7. converted the text of the Italian tweets into English using ‘googletrans’ tool [12].

In the second part, a higher quality dataset was required for the topic model. The duplicate tweets were removed, and only the unique tweets remained. Apart from the general data cleaning methods, tokenization and lemmatization could enable the model to achieve better performance.

We tokenized the text using Gensim library [13] and converted all the text content to lowercase to ensure every word appears in a consistent format. Then we pruned the vocabulary, removing stop words and removing terms unrelated to the topic and we made a bigrams model. Finally, the spaCy library of NLTK [14] was used to accomplish lemmatization.

### 2.3 Sentiment analysis

Humans associate words, phrases, and sentences with emotion, and Sentiment analysis uses computational algorithms to extract and measure the emotion expressed within a text. In the present study, the Valence Aware Dictionary and sEntiment Reasoner (VADER) model was used to decode the sentiments, opinions, evaluations and emotions regarding COVID-19 vaccines within collected Tweets.

Introduced in 2014 by Hutto and Gilbert [9] and designed with a focus on social media texts, VADER is a pre-trained gold standard sentiment lexicon and rule-based sentiment analysis tool that works exceedingly well in microblog-like contexts, where by the short-text data is a complex mix of a variety of features and a sentence may contain multiple sentiments all at once. VADER Sentiment Analysis is free available as a vaderSentiment module incorporated into the Python NLTK package and can be applied directly to unlabelled text data. The reasons for using VADER in our study are manifold and lie in its many advantages over traditional methods of Sentiment Analysis, including:

- It does not require any training data. It is constructed from a crowd-validated gold standard sentiment lexicon (along with the associated sentiment intensity measures), which is specifically attuned to sentiment in microblog-like contexts like Twitter
- It has been shown to achieve excellent classification accuracy scores in assessing the sentiment of Tweets, outperforming individual human raters and performing as well as (and in most cases, better than) other highly regarded sentiment analysis tools [9]
- It is computationally fast and does not suffer overmuch from a speed-performance trade-off.

Based on its complete rules, VADER can carry out sentiment analysis on various lexical features: punctuation, capitalization, degree modifiers, the contrastative conjunction ‘but’, negation flipping tri-gram.

The sentiment score of a sentence is calculated by summing up the lexicon rates of each VADER-dictionary-listed word in the sentence. After applying a proper normalization, VADER returns a ‘compound’ sentiment score (*S*_*s*_) in the range −1 to 1, from most negative to most positive. Once the score *S*_*s*_ is known, threshold values can be used to categorise tweets as either positive, negative, or neutral.

The VADER model returns the sentiment score and allows us, through the appropriate thresholds, to classify each tweet by its polarity: negative, positive or neutral (see Table 1).

**Table 1:** Compound sentiment score and polarity.

S_*s*_	Polarity
*S*_*s*_ > +0.05	Positive
−0.05 ≤ *S*_*s*_ ≤ +0.05	Neutral
*S*_*s*_ < −0.05	Negative

### 2.4 Topic modeling

Topic Modelling is an unsupervised machine learning technique that aims to identify topics that best describe the contents of a set of documents. It is a text mining procedure with which the themes of documents can be identified from a large collected document corpus [15]. The Latent Dirichlet Allocation (LDA) model is one of the most popular topic modelling methods. It is a probabilistic model for expressing a corpus based on a three-level hierarchical Bayesian model. The basic idea of the LDA is that each document has a topic, and a topic can be defined as a word distribution [16]. Particularly in LDA models, the generation of documents within a corpus follows the following process:

1. A mixture of *k* topics, *θ*, is sampled from a Dirichlet prior, which is parameterized by *α*;
2. A topic *z*_*n*_ is sampled from the multinomial distribution, *p*(*θ* ∣ *α*) that is the document topic distribution which models *p*(*z*_*n*_ = *i* ∣ *θ*);
3. Fixed the number of topics *k* = 1…, *K*, the distribution of words for *k* topics is denoted by *ϕ*, which is also a multinomial distribution whose hyper-parameter *β* follows the Dirichlet distribution;
4. Given the topic *z*_*n*_, a word, *w*_*n*_, is then sampled via the multinomial distribution *p*(*w* ∣ *z*_*n*_; *β*).

Overall, the probability of a document (or tweet, in our case) “**w**” containing words can be described as:

$$\begin{array}{c} p\left(w\right)=\int_{\theta }p\left(\theta |\alpha \right)(\prod_{n=1}^{N}\sum_{z_{n}=1}^{k}p\left(w_{n}|z_{n};\beta \right)p\left(z_{n}|\theta \right))d\theta \end{array}$$ (1)

Finally, the probability of the corpus of *M* documents *D* = {**w**_1_, …, **w**_M_} can be expressed as the product of the marginal probabilities of each single document *D*_*m*_, as shown in (2).

$$\begin{array}{c} p\left(D\right)=\prod_{m=1}^{M}\int_{\theta }p\left(\theta_{m}|\alpha \right)(\prod_{n=1}^{N_{m}}\sum_{z_{n}=1}^{k}p\left(w_{m,n}|z_{m,n};\beta \right)p\left(z_{m,n}|\theta_{m}\right))d\theta_{m} \end{array}$$ (2)

In our analysis. which includes four months of tweets, we find that the content of tweets is changeable over time, and thus that the topic content is not a static corpus. The Dynamic Latent Dirichlet Allocation (DLDA) is adopted and used on topics aggregated in time epochs, and a state-space model handles transitions of the topics from one epoch to another. A gaussian probabilistic model to obtain the posterior probabilities on the evolving topics along the timeline is added as an additional dimension.

**Fig 1:**
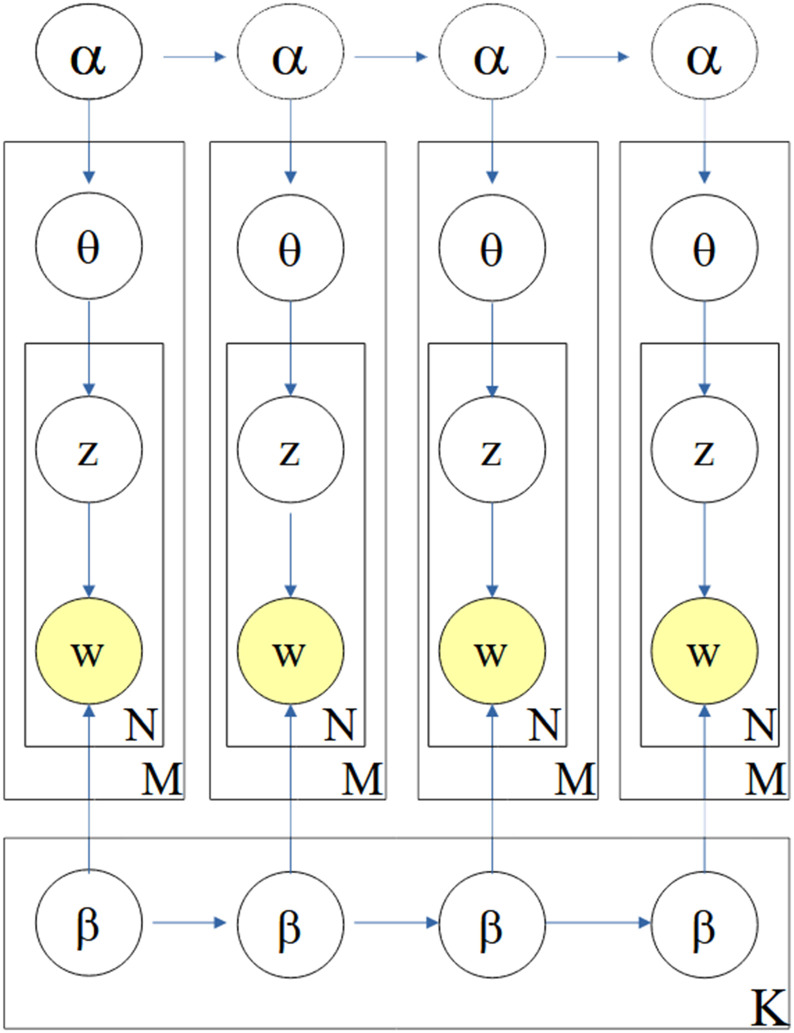
Dynamic topic model (for four time slices). A set of topics in the dataset are evolved from the set of the previous slice over time series. The model for each time slice corresponds to the original LDA process. Additionally, each topic’s parameters and evolve over time. (Blei and Lafferty, 2006 [10]).

Fig 1 shows a graphical representation of the Dynamic Topic Model (DTM) [10]. As a part of the probabilistic topic models class, the dynamic one can catch how the various themes of tweets evolved. The tweets dataset corpus used here (February-May, 2021) contains 4 months. The dynamic topic model is accordingly applied to four-time steps corresponding to the four months of the data set. These time-slices are put into the model provided by gensims [13].

An essential challenge in DLDA (as LDA) is to determine an appropriate number of topics. Roder et al. [17] proposed coherence scores to evaluate the quality of each topic model. Particularly, topic coherence is the measure to evaluate the coherence between topics inferred by a model. As coherence measures, we have used *C*_*v*_ that is a measure based on a sliding window that uses normalized point-wise mutual information (NPMI) and cosine similarity.

These values aim to emulate the relative score that a human is likely to assign to a topic and indicate how much the topic words ‘make sense’. These scores infer the cohesiveness between ‘top’ words within a given topic. Besides, the distribution on the primer component analysis (PCA) is considered, which can visualize the topic models in a word spatial with two dimensions. A uniform distribution is preferred, which is considered a high degree of independence for each topic. The judgment for a good model is a higher coherence and an average distribution on the primer analysis displayed by the pyLDAvis [18].

## 3 Results

### 3.1 Exploring the COVID-19 vaccine tweets

**Fig 2:**
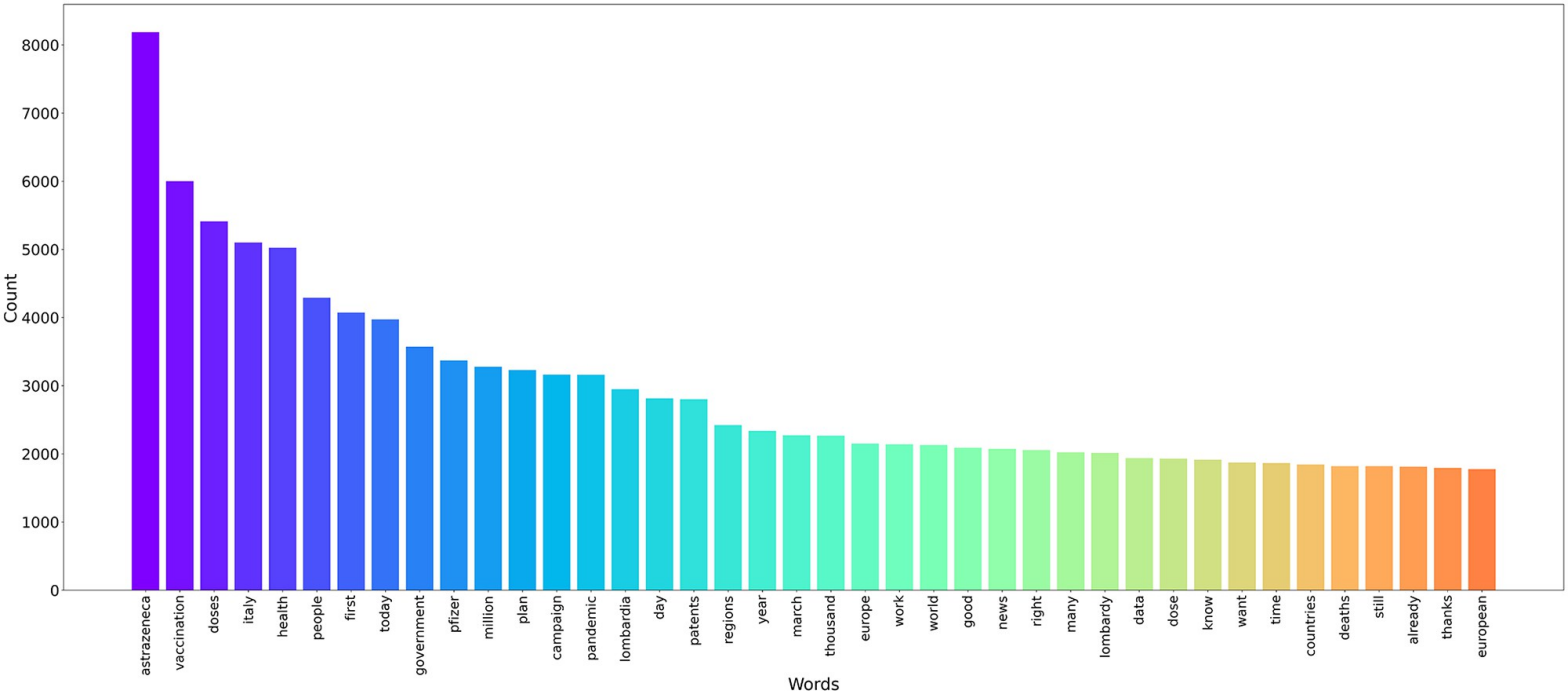
The total text word frequency. After removing irrelevant words, word frequency of main opinion is counted and visualized.

The word frequency of the most frequent 40 words terms are counted and visualized in Fig 2. The only word of concern quoted is ‘deaths’ due to some cases of deaths that the media had attributed to some batches of ‘Oxford-AstraZeneca’ vaccines, but the most words references to the number of doses available and to the progress of the vaccination campaign.

**Fig 3:**
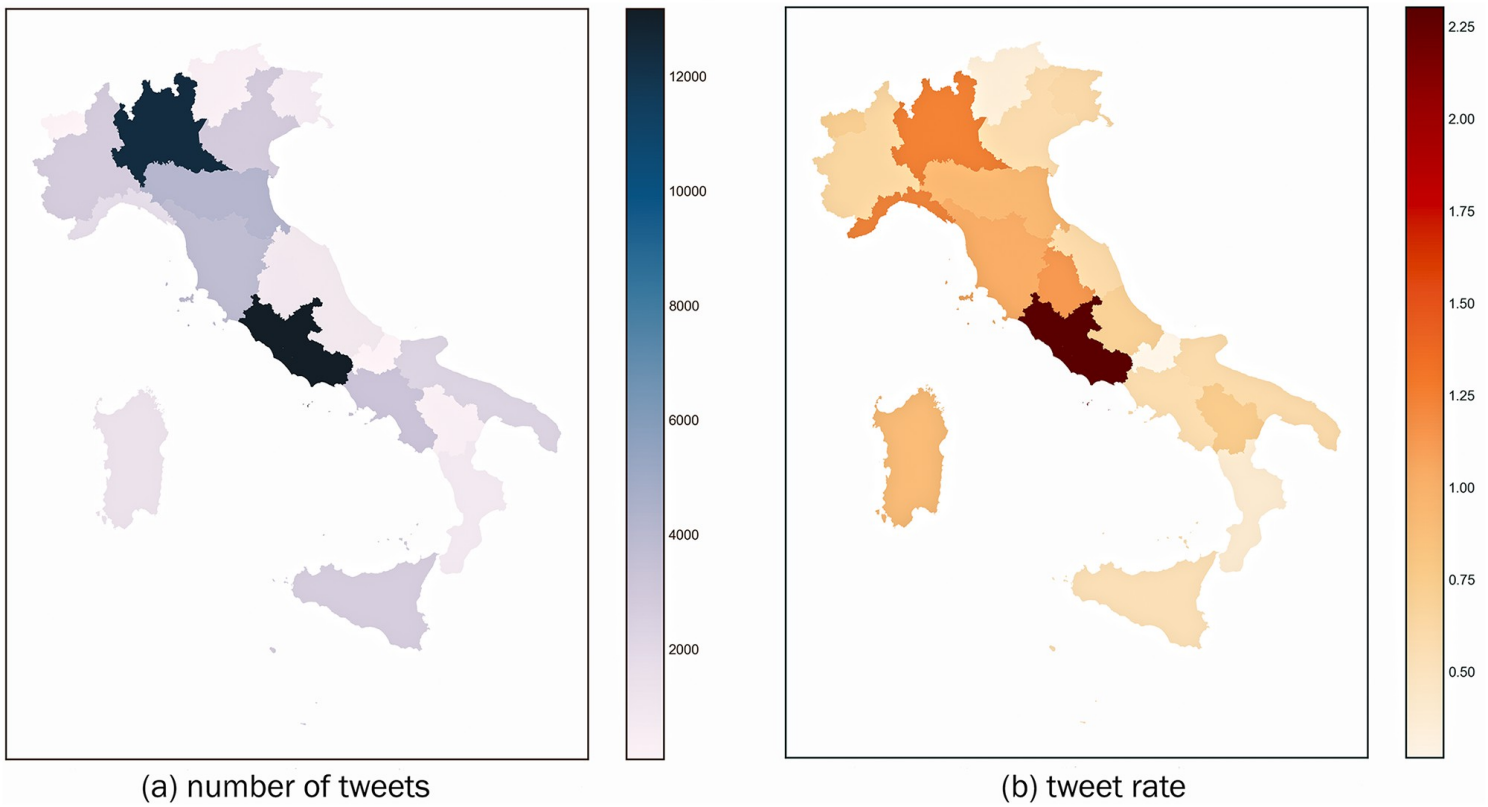
Number of tweets for Italian regions: Absolute values (a) and tweet rate (b).

The ‘location’ features detected in approximately 18,000 correctly localised tweets highlights the number of tweets in the 20 Italian regions. The regions with the highest number of tweets are Lazio and Lombardy, that are also the most populated (Fig 3a). Fig 3b shows the ‘tweet rate’ of each region, that is the number of tweets per 1,000 residents: Lazio is the region with the highest rate, closely followed by Lombardy and Liguria.

### 3.2 Sentiment analysis

The output of the VADER model shows a moderate predominance of negative public opinion (Table 2). As an example, three tweets with their own polarity are shown in the Table 3.

**Table 2:** Number of tweets for sentiment polarity.

SP	absolute values	percentages
Positive	25849	35.1
Negative	26608	36.1
Neutral	21231	28.8

**Table 3:** Some examples of tweets with sentiment score (S_*s*_) and polarity.

polarity	S_*s*_	Text
Negative	-0.9566	THREE DOCTORS DEAD (Maybe they were vaccinated?) But the government does not open a commission to investigate Vaccines? ** PEOPLE DIE ** Doctor killed by Covid in the Canavese, is the third in a week in Piedmont
Neutral	0	YouTrend regional index shows that Veneto is further ahead in the vaccination campaign with 84 points out of 100, followed by Lombardia and Puglia at 82
Positive	0.9618	The only program that shows the best side of the vaccines, is the happiness of the mostly elderly people who have received the dose and welcome with relief and hope

In Italy, the most talked about COVID-19 vaccines brands were Oxford-AstraZeneca (66.1%) and Pfizer-BioNTech (14.89%) see Table 4.

**Table 4:** Sentiment analysis result of tweets towards vaccine brands.

Vaccine brand	*n*_*pos*_	*n*_*neg*_	*n*_*neu*_	*n*_*tot*_	*n*_*tot*_(%)
Oxford-AstraZeneca	1964	2291	1455	5710	66.51%
Johnson & Johnson	248	204	282	734	8.55%
Moderna	48	46	83	177	2.06%
Pfizer-BioNTech	391	463	424	1278	14.89%
Sputnik-V	256	236	194	686	7.99%

Columns after the first indicate respectively: the number of positive, negative, neutral and total tweets with their percentages.

**Fig 4:**
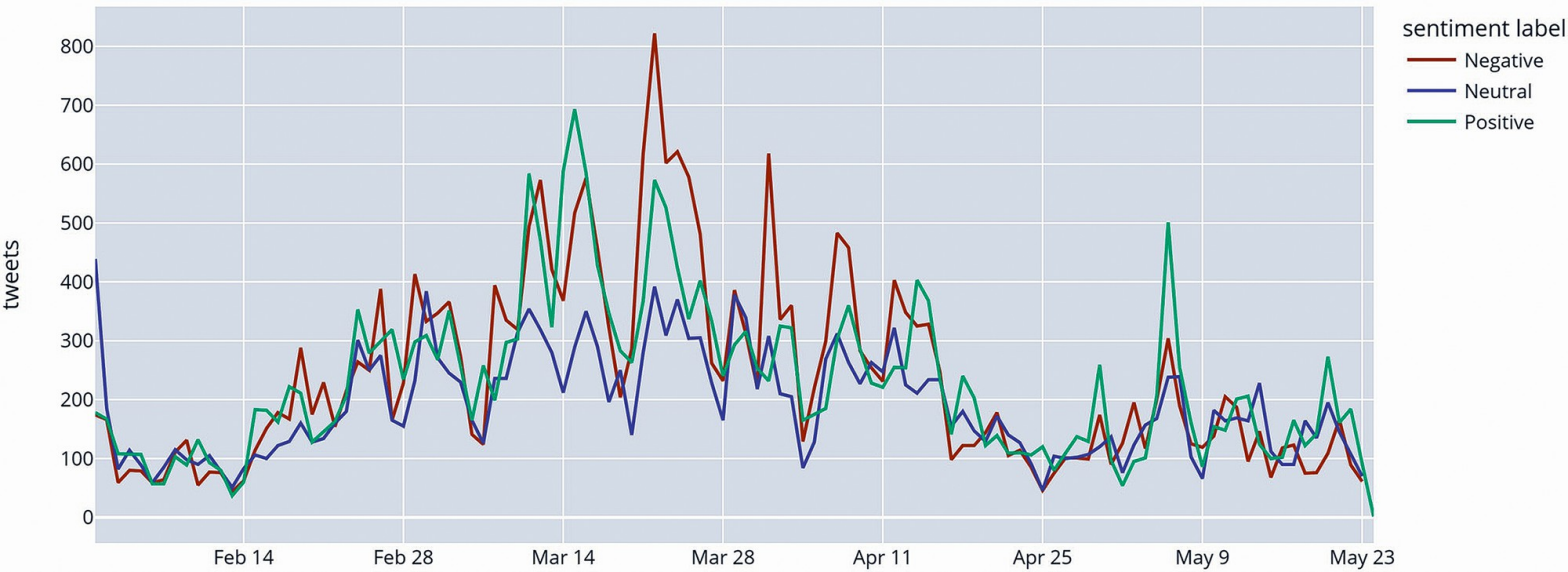
Timeline showing sentiment of tweets about COVID-19 vaccines.

Fig 4 shows the trend in the number of daily tweets. The peak in the second half of March corresponds to the temporary suspension of AstraZeneca inoculations due to the supposed relation between the jab and some cases of death.

Even after the positive verdict of the European Medicines Agency (EMA) eight days after the stop, the climate of uncertainty [19] continued to impact on the vaccination campaign, leading to the phenomenon of vaccine hesitation. This meant reluctance, delay and indecision about being vaccinated out of fear of having adverse reaction to the shot, eventually leading to increased distrust about vaccines and pharmaceutical regulation institutions.

**Fig 5:**
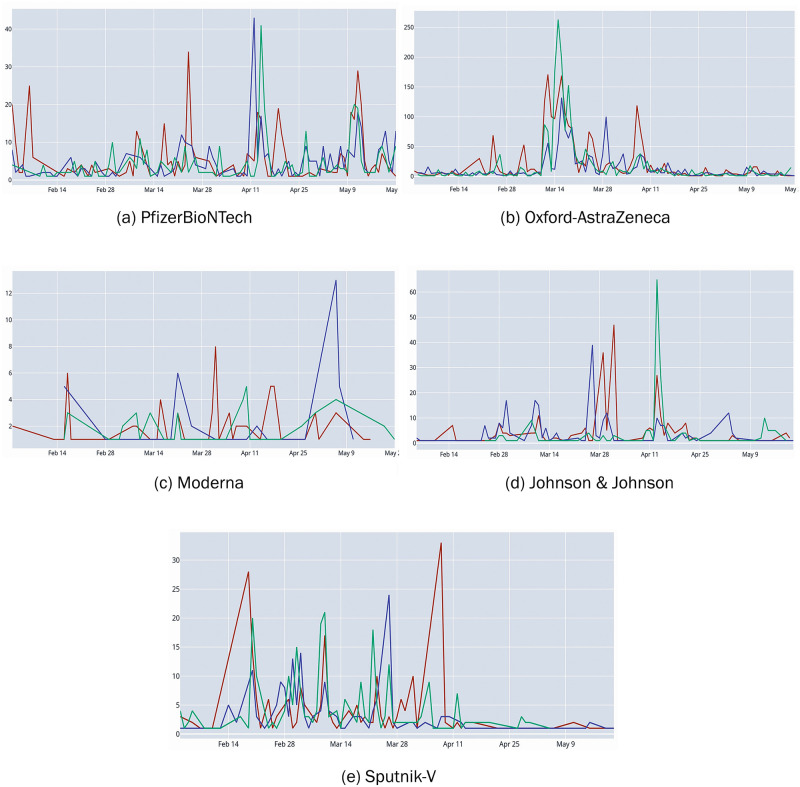
Timeline showing sentiments of tweets about different vaccine brands. Each graph reports the daily number of tweets for the observed period. Different line colours indicate tweet polarity: green (positive), red (negative) and blue (neutral).

As it is possible to see in the timeline of sentiments about vaccine brands (Fig 5), the suspension of AstraZeneca administrations brought negative opinions on all the other brands except for Sputnik-V. The latter brand has never been available in Italy, despite its approval in other 18 extra-EU countries [20].

Moreover, just in February 2021, the Lancet journal published the results of a study on the efficacy of the Sputnik-V vaccine [21]: the study showed that the jab was 91.6 per cent effective at preventing infection, a much higher value compared to that of Oxford-AstraZeneca (70.4 per cent), published in the same journal two months earlier [22]. Even on severe side effects, Sputnik outperfomed AstraZeneca: 0.30 per cent against 0.69 per cent. Therefore, a possible reading of the sentiment analysis results is that Italians preferred Sputnik over AstraZeneca for its greater effectiveness and the lower percentage of severe side effects.

**Fig 6:**
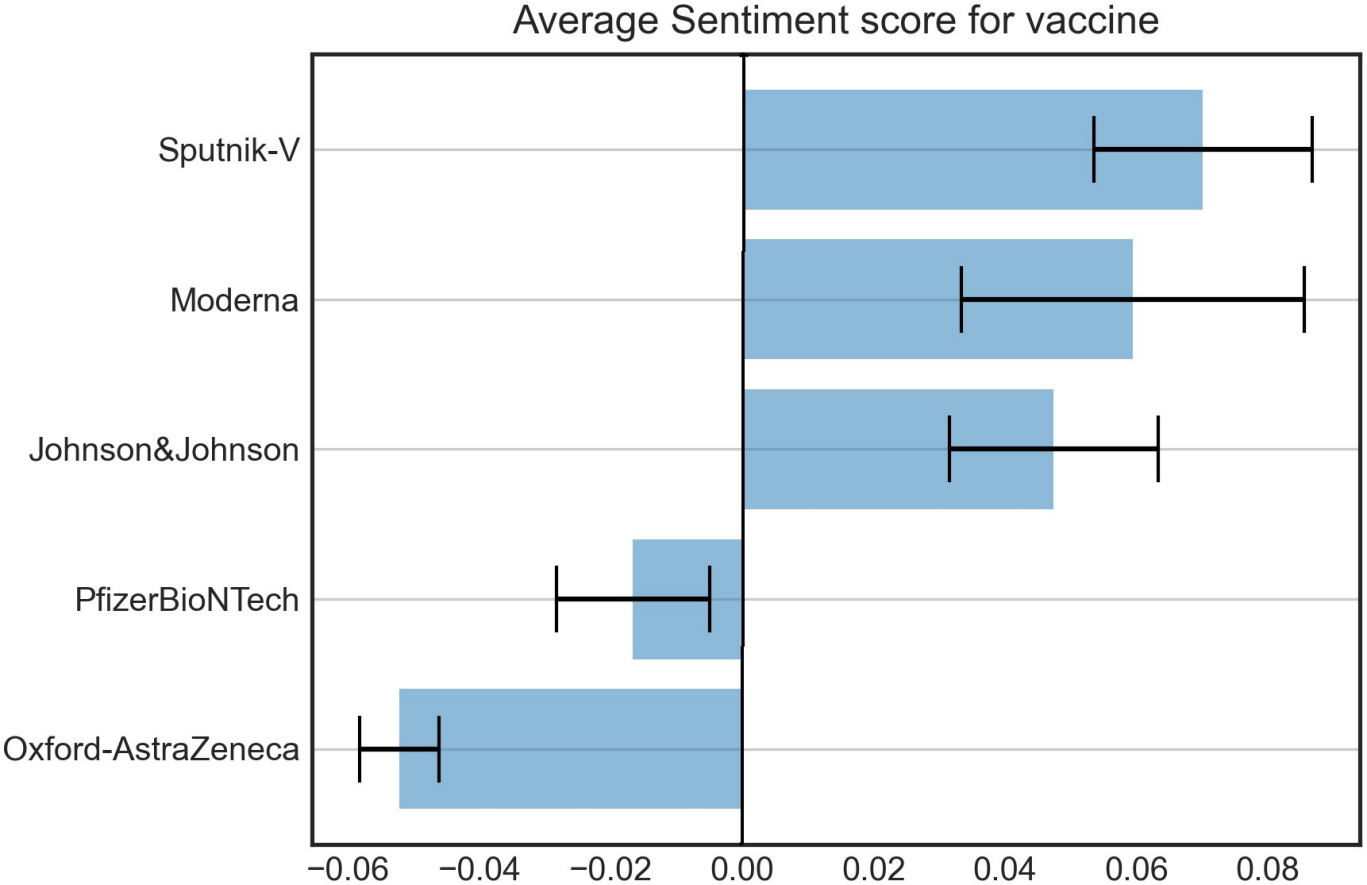
Average sentiment score by COVID-19 vaccines brand. The error bar indicates the relative standard error from the mean.

AstraZeneca presents the highest negative average sentiment score, with a small standard error from the mean, while Sputnik-V shows the highest positive average score, with a greater standard error than AstraZeneca (Fig 6).

**Fig 7:**
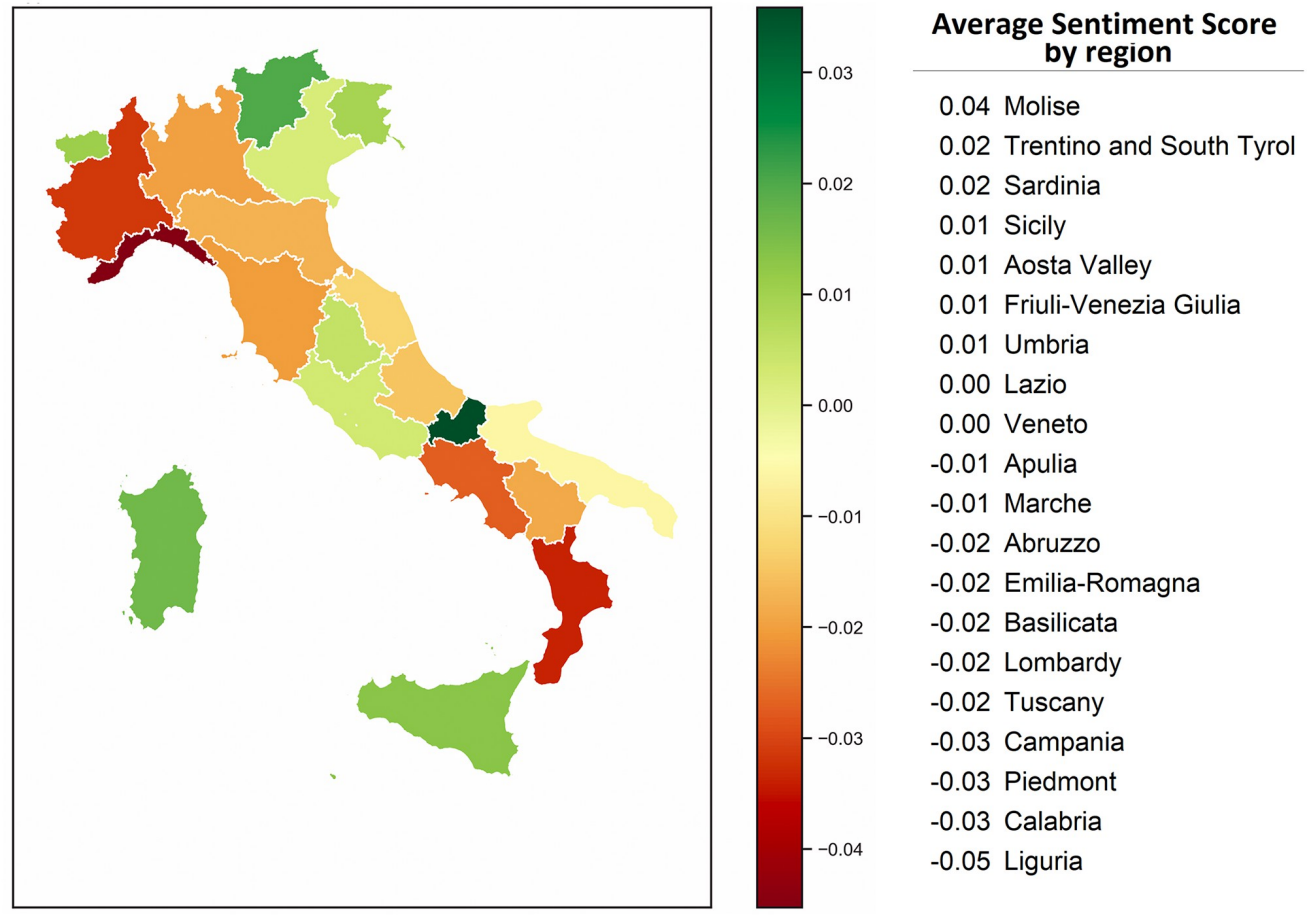
Average sentiment score in Italy by region.

The analysis carried out at the regional level was performed only on 18,000 tweets that had a regional geolocation. Fig 7, shows the average sentiment scores of the Italian regions: the sentiment score is neutral (between -0.05 and +0.05, see Table 1) for all regions. Indeed, there are no major differences in vaccination campaign in Italy from region to region. Furthermore, the result is consistent with the flattening due to the use of the average of the scores.

### 3.3 The topic model

To explore what the user is concerned about on Twitter with reference to COVID-19 vaccines, we applied the LDA model to our clean corpus, taking only the following tagger components: nouns, adjectives, verbs and adverbs.

To determine the appropriate number of topics, *C*_*v*_ [17] was used as a measure of topic coherence, that is the coherence between topics inferred by the model (see par. 1.4). Besides, the distribution on the primer component analysis (PCA) is considered, which can visualize the topic models in a word spatial with two dimensions. A uniform distribution is preferred, which is considered a high degree of independence for each topic. The judgment for a good model is a higher coherence and an average distribution on the primer analysis displayed by the pyLDAvis [18].

**Fig 8:**
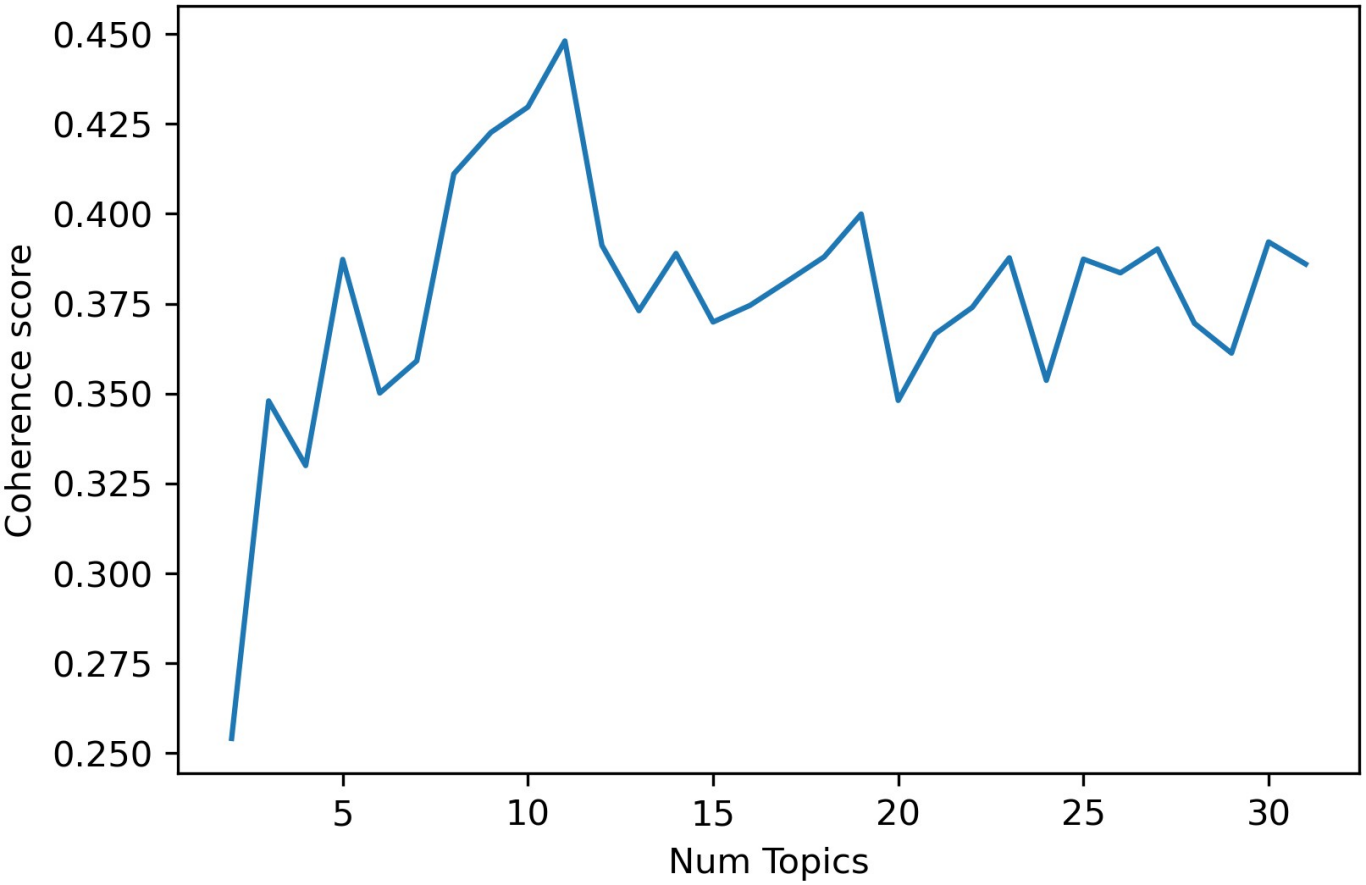
Coherence values.

By using topic numbers *k* ranging from 2–30, we initialised the LDA models and calculated the model’s coherence. According to Fig 8, the coherence score peaked at 3, 5 and 11 topic numbers. The choice of 5 or 11 topic numbers would lead to a nonuniform distribution on primer component analysis (PCA), which means that there is not a high degree of independence for each topic (see LDAvis interactive map in S1–S3 Files). Therefore, we chose 3 as the topic number: the model has no intersections among topics, summarizes the whole word space well, and the topics remain relatively independent [23].

**Fig 9:**
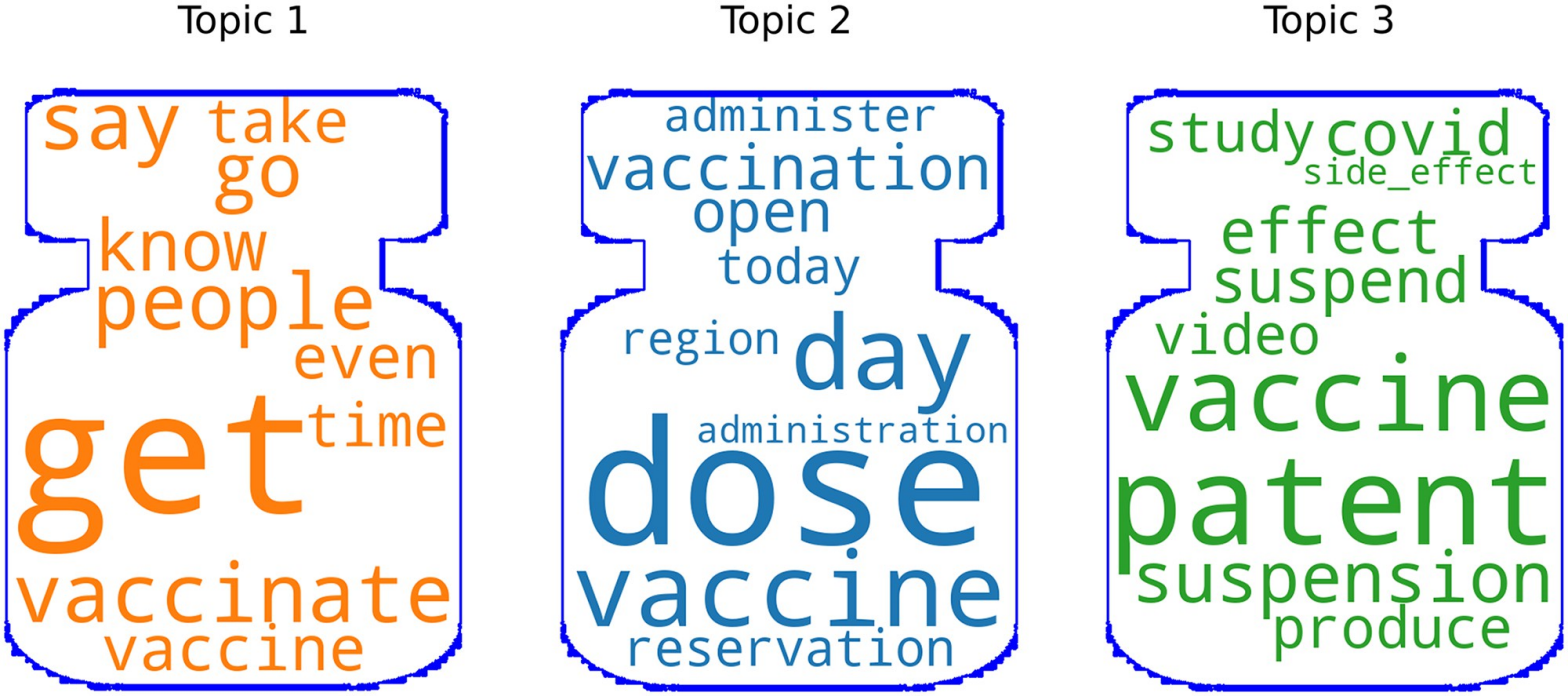
Wordclouds: Top words in each topic cluster.

The LDA analysis is shown in Table 5. The first theme takes up to 45% of total tokens and includes tokens such as ‘people’, ‘get’, ‘vaccinate’. Based on this, we inferred that most people showed interested in the vaccination campaign and wanted to be vaccinated. The second theme takes up to 30% of the total tokens, including the words ‘day’, ‘vaccine’, ‘dose’ and ‘administration’ showing people’s interest in vaccination plan. Apart from that, several words, like ‘effect’, ‘health’, ‘suspension’ and ‘patent’, are mentioned in the third topic. This indicates concerns about vaccination measures and side effects. See Fig 9 for a word cloud representation of the three topics and the S1 File for their interactive map.

**Table 5:** LDA results: Percentage size and first 15 words of each topic.

	Topic 1 (45%)	Topic 2 (30%)	Topic 3 (25%)
1	get	dose	patent
2	vaccinate	vaccine	vaccine
3	say	day	suspension
4	people	vaccination	covid
5	go	open	effect
6	know	reservation	sospend
7	vaccine	today	produce
8	even	administer	study
9	take	ragion	video
10	time	administration	side_effect
11	want	book	production
12	make	first	vacation
13	good	start	health
14	give	year_old	word
15	talk	campaign	case

The LDA model output identified the following three topics:

- Topic 1: Vaccination plan info: such as doses, administrations, the regions concerned
- Topic 2: Usefulness of vaccinating: a prevalent positive outlook
- Topic 3: Concerns about vaccines: risks, side effects, safety.

**Fig 10:**
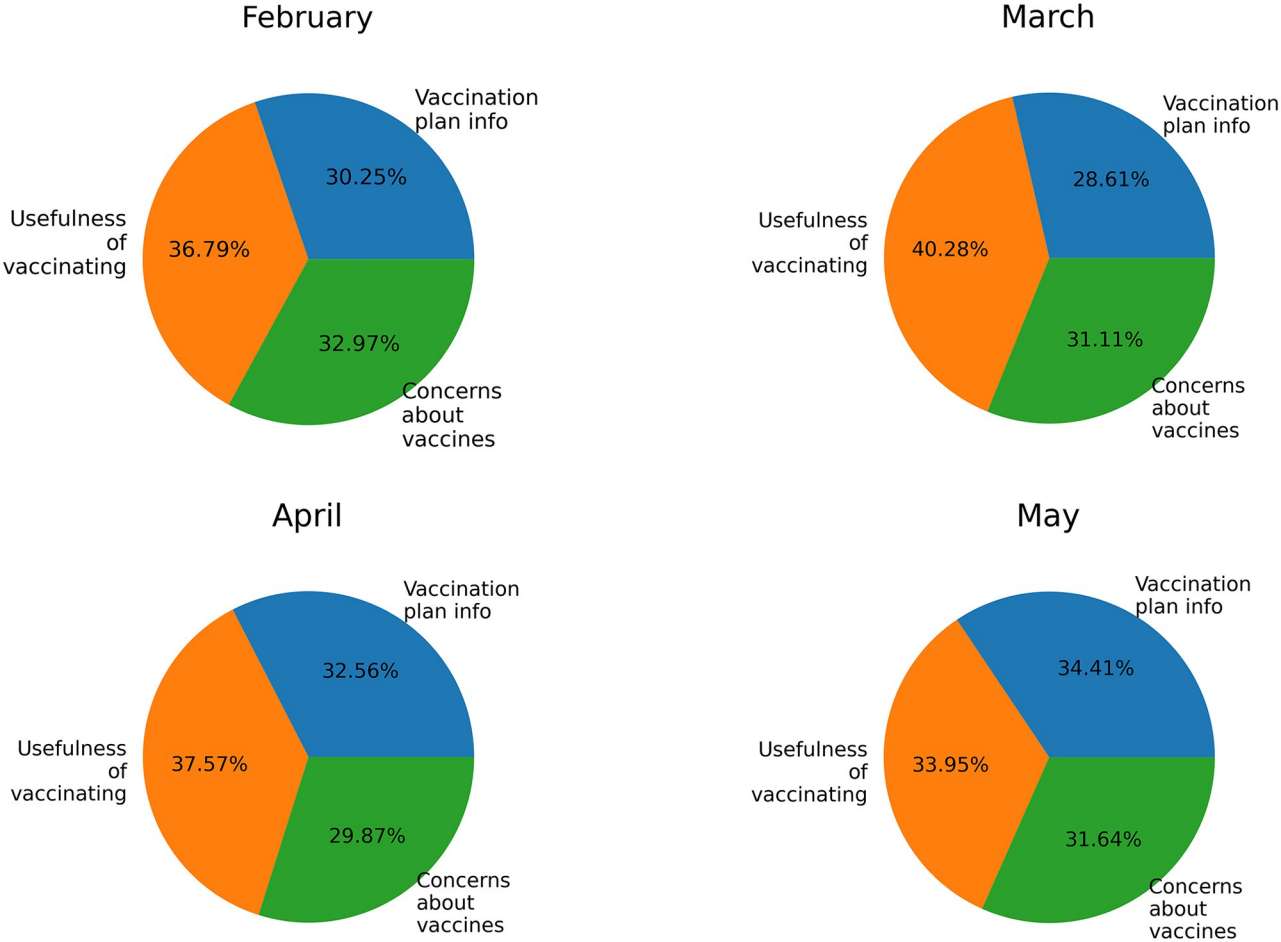
Dynamic volume of each topic over time.

The pie charts in Fig 10 shows the dynamic volume of each topic in the four periods. It is worth noting that the topics remained quite stable over the months except for topic 2 (usefulness of vaccinating) that grew slightly in the second month and then decreased in the next months.

**Fig 11:**
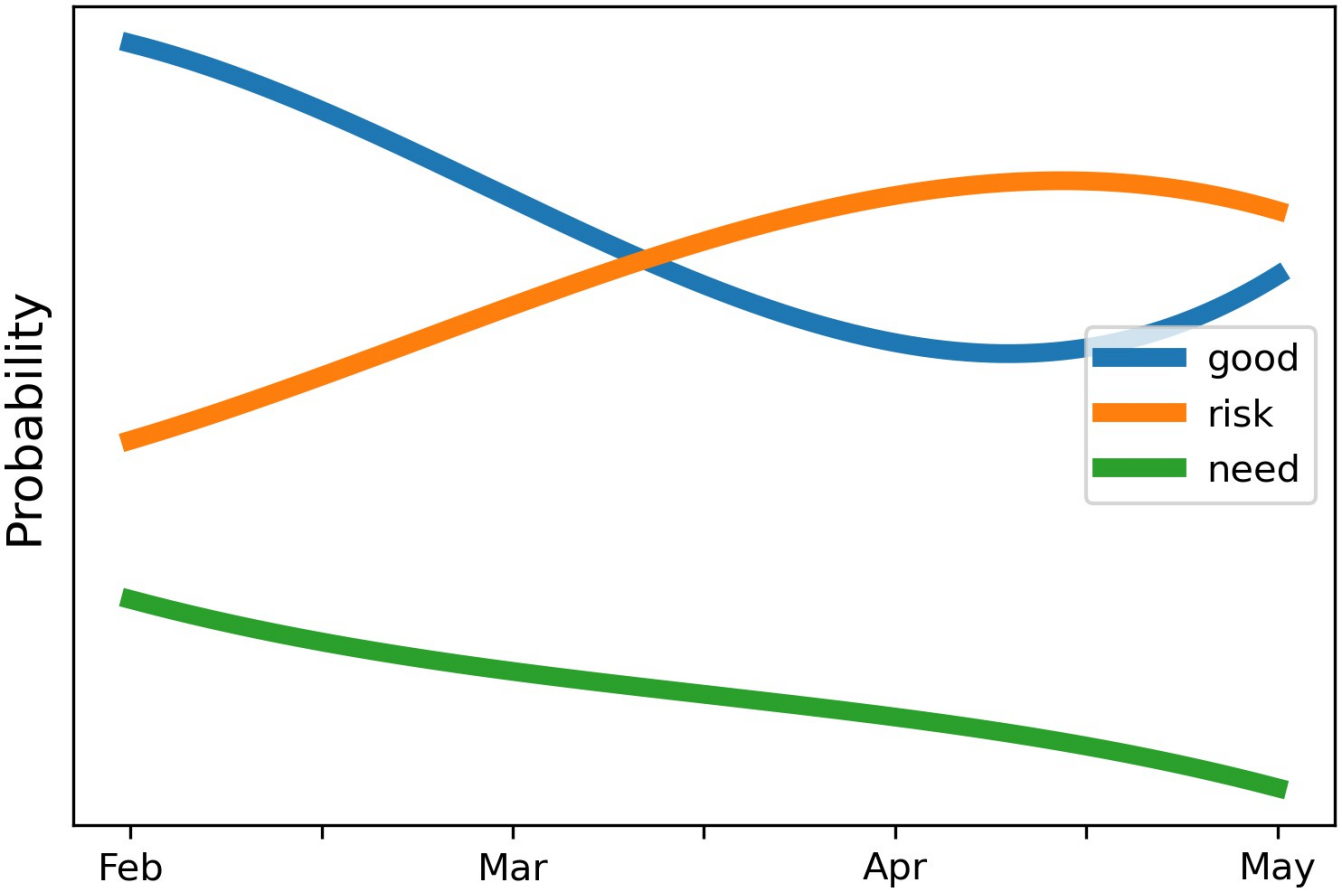
Dynamic topic model: Probabilities of some notable terms over the 4 months.

Fig 11 plots the probabilities of some notable terms (the most probable words in the topics predicted by the DTM) that change direction over the period. During the first four months of the vaccination campaign, attributions referring to vaccines such as ‘good’ and ‘need’ decreased their probability in the model. That decrease happened at the same time of the suspension of AstraZeneca brand throughout Italy [24] in order to make checks in relation to the deaths of two men [25]. A few days later, AIFA revoked the ban and vaccinations resumed. This could explain the slight rise in use of the term ‘good’ occurring between April and May. However, after these events, people began to have doubts, not only about the effectiveness of vaccines, but also about the side effect risks of vaccination and the necessity to be vaccinated or not. In our opinion, this could be the reason why the probability of the term ‘risk’ progressively increases over the months, while, on the other hand, the probability of the term ‘need’, which expresses people’s need to be vaccinated, slowly decreased (Fig 11).

## 4 Limitations

This study has some limitations. First of all, we only focused on Italian tweet contents. However, users may be distributed among different social media platforms and different countries according to their usage, language, and preferences. Anyway, the methods used in our study can be extended to different social media platforms.

It is worth noting that the most recent statistics about social media usage show that approximately 83% of Twitter users worldwide were under age 50 [26]; this implies that Twitter-based studies generally suffer from an underestimation bias in the opinions of people aged 50 and over.

Additionally, Padilla et al. [27] found geographical bias in their analysis of Twitter data and found an overrepresentation of urban areas in the demographic data of Twitter users included in their study. Given this prior research, we must assume that users from urban areas are overrepresented in this data set as well.

The VADER model used for sentiment analysis uses a lexical approach. That means it uses words or vocabularies that have been assigned predetermined scores as positive or negative. The scores are based on a pre-trained model labeled as such by human reviewers. Therefore, there are also some disadvantages to this approach:

- Misspellings and grammatical mistakes may cause the analysis to overlook important words or usage.
- Sarcasm and irony may be misinterpreted.
- Discriminating jargon, nomenclature, memes, or turns of phrase may not be recognized.

Regarding topic analysis, considering unsupervised learning such as LDA, the primary limitation is some degree of subjectivity in defining the topic created [28].

Duplicated Tweets Bots posting on Twitter are a well-documented phenomenon [29, 30]. One of the issues our study faced was the duplication of content due to bot activity on the topic of vaccines. Other research has documented bot activity on COVID-19 and COVID-19 vaccine misinformation as well [29, 31]. The main issue this may cause in our analysis is that bot activity may overinflate the importance of certain topics. To combat this, we removed retweets and duplicate tweets in topic analysis: the number of tweets from initial 73 thousand reduced to approximately 26 thousand tweets in our topic analysis.

## 5 Discussion and conclusions

The current COVID-19 pandemic has resulted in a surge of social media use as a forum for discussing an array of topics about the pandemic, including vaccines. However, social media users can be exposed to negative sentiments and misinformation [32], which may influence individual views and lead to vaccine hesitancy or refusal [33].

Currently, little is known about public opinion regarding COVID-19 vaccines in Italy. In particular, identifying vaccine hesitancy opinions is of great importance as the refusal to take anti-Covid vaccinations is concerning from a public health point of view.

To the best of our knowledge, this one the first study on Twitter to identify opinions about COVID-19 vaccination in Italy and their progression over the first months of the vaccination campaign. Moreover, better understanding public opinion by categorizing of the Twitter content is useful to identify how the vaccine hesitancy phenomenon was born in Italy. Our results can help policymakers and research communities tracking public attitude towards COVID-19 vaccines and helping them make decisions to promote the vaccination campaign.

In our study most sentiments towards COVID-19 vaccines were negative (36.1%) or positive (35.1%) and only 28.8% neutral (Table 2). The answers to our three main questions related to the vaccination campaign in Italy are as follows:

- The overall sentiment towards COVID-19 vaccines showed a slight predominance of people with negative attitudes. At the same time, the analysis conducted in the Italian regions showed an average neutral sentiment score in all the areas. In our opinion, these data indicates uniformity of the vaccination campaigns on the different Italian areas.
- In Italy, the most talked about COVID-19 vaccines brands were Astrazeneca and Pfizer-BioNTech, which concerned respectively the 66.13% and the 15.06% of total tweets. Among vaccine brands, Italian people preferred Sputnik-V, even though they had not tried it, and disliked AstraZeneca.
- Three topics were discovered to be most popular in vaccines tweets. These themes remained quite stable over time and respectively represent: (1) Vaccination plan info, (2) Usefulness of vaccinating and (3) Concerns about vaccines. Over the course of the months, people began to have doubts not only about the goodness of vaccines, but with respect to the risks of vaccination and the necessity to be vaccinated.

In conclusion, our analysis highlighted how fluctuations in opinions of COVID-19 vaccines and the evolution of topics have been affected by news regarding vaccine side effects. Also, the opinions towards the different brands of vaccines have been determined in the same way by the news regarding the effectiveness and possible severe side effects.

Our outcomes of sentiment categories and the topics identified from Twitter are in line to that of some studies, always referred to the first months of starting of the vaccination campaign in other countries.

In the US, a study about the vaccines on social media, confirm our results [34]. Through the trend analysis, it was found that the peaks of the topics were impacted by the events reported in the news and spread through social media. The sentiment analysis showed that 46.9% of the tweets were negative, 33.2% of tweets were positive and 19.9% of tweets were neutral. At the same time the topic analysis found that the administration and access to vaccines were some of the major concerns. A study conducted by Fazel et al. [35] in the United Kingdom confirmed a predominance of tweets with negative vaccine content that varied according to major news announcement. On the contrary, a research on COVID-19 vaccines that focused on tweets in English all over the world, revealed that the dominant sentiments were positive and neutral [36], but the main topics were always vaccine information and knowledge, vaccine hesitancy and severe side effects of the vaccines.

Future studies could investigate how perceptions and opinions about Covid-vaccines will change in the coming months and years, using sources other than Twitter and combining results of different European countries. Furthermore, our model can be extended to other research problems such as identifying misinformation on social media or to train a topic model with LDA to forecast event topics and trends.

## Supporting information

S1 File Visualization for LDA topic modelling with 3 topic number. (HTML)

S2 File Visualization for LDA topic modelling with 5 topic number. (HTML)

S3 File Visualization for LDA topic modelling with 11 topic number. (HTML)

## References

[1] Johns Hopkins Coronavirus Resource Center. COVID-19 map[Online]; 2021. Available from: https://coronavirus.jhu.edu/map.html.

[2] Bank of Italy. The impact of the COVID-19 pandemic on the italian economy: illustrative scenarios; 2021. Available from: https://www.bancaditalia.it/pubblicazioni/note-covid-19/2020/Scenarios_impact_COVID_19.pdf?language_id=1.

[3] Ministry of Health. Piano vaccini anti Covid-19; 2021. Available from: https://www.salute.gov.it/portale/nuovocoronavirus/dettaglioContenutiNuovoCoronavirus.jsp?lingua=italiano&id=5452&area=nuovoCoronavirus&menu=vuoto.

[4] Kemp S. Digital 2020: Global Digital Overview; 2020. Online. Available from: https://datareportal.com/reports/digital-2020-global-digitaloverview.

[5] Zhan Y, Etter JF, Leischow S, Zeng D. Electronic cigarette usage patterns: a case study combining survey and social media data. J Am Med Inform Assoc. 2019;26(1):9–18. doi: 10.1093/jamia/ocy140 30544163 PMC6308011

[6] Tumasjan A, Sprenger T, Sandner P, Welpe I. Predicting Elections with Twitter: What 140 Characters Reveal about Political Sentiment. In: Proc. Fourth Int. AAAI Conf. Weblogs Soc. Media Predict. vol. 10; 2010.

[7] Chew C, Eysenbach G. Pandemics in the age of Twitter: content analysis of Tweets during the 2009 H1N1 outbreak. PLoS One. 2010;5(11):e14118. doi: 10.1371/journal.pone.0014118 21124761 PMC2993925

[8] Hassanpour S, Tomita N, DeLise T, Crosier B, Marsch LA. Identifying substance use risk based on deep neural networks and Instagram social media data. Neuropsychopharmacology. 2019;44(3):487–494. doi: 10.1038/s41386-018-0247-x 30356094 PMC6333814

[9] Hutto CJ, Gilbert E. VADER: A Parsimonious Rule-based Model for Sentiment Analysis of Social Media Text; 2015.

[10] Blei DM, Lafferty JD. Dynamic topic models. In: Proceedings of the 23rd international conference on Machine learning—ICML’06. New York, New York, USA: ACM Press; 2006.

[11] TrackMyHashtag. Social media analytics tool which can track all the activities happening around a Twitter campaigns. https://www.trackmyhashtag.com.

[12] Googletrans web version for documents; 2021. Google LLC. https://translate.google.it/?sl=it&tl=en&op=docs.

[13] Řehůřek R, Sojka P. Software Framework for Topic Modelling with Large Corpora. In: Proceedings of LREC 2010 workshop New Challenges for NLP Frameworks. Valletta, Malta: University of Malta; 2010. p. 46–50.

[14] Bird S, Klein E, Loper E. Natural language processing with python. Sebastopol, CA: O’Reilly Media; 2009.

[15] Blei DM, Ng AY, Jordan MI, Lafferty J. Latent dirichlet allocation. Journal of Machine Learning Research. 2003;3:993–1022.

[16] Lee J, Kang JH, Jun S, Lim H, Jang D, Park S. Ensemble modeling for sustainable technology transfer. Sustainability. 2018;10(7):22–78. doi: 10.3390/su10072278

[17] Röder M, Both A, Hinneburg A. Exploring the space of topic coherence measures. New York, New York, USA: ACM Press; 2015.

[18] Sievert C, Shirley K. LDAvis: A method for visualizing and interpreting topics. In: Proceedings of the Workshop on Interactive Language Learning, Visualization, and Interfaces. Baltimore, Maryland, USA: Association for Computational Linguistics; 2014. p. 63–70. Available from: https://aclanthology.org/W14-3110.

[19] Petersen MB, Jørgensen F, Lindholt MF. Did the European suspension of the AstraZeneca vaccine decrease vaccine acceptance during the COVID-19 pandemic? Vaccine. 2022;40(4):558–561. doi: 10.1016/j.vaccine.2021.12.026 34952752 PMC8693776

[20] STATISTA. Major COVID-19 vaccines by number of countries where they are used as of March 2021; 2021. Statista. https://www.statista.com/statistics/1223436/covid-19-vaccines-by-number-of-countries/.

[21] Jones I, Roy P. Sputnik V COVID-19 vaccine candidate appears safe and effective. The Lancet. 2021;397(10275):642–643. doi: 10.1016/S0140-6736(21)00191-4 33545098 PMC7906719

[22] Knoll MD, Wonodi C. Oxford–AstraZeneca COVID-19 vaccine efficacy. The Lancet. 2021;397(10269):72–74. doi: 10.1016/S0140-6736(20)32623-4 33306990 PMC7832220

[23] Syed S, Spruit M. Full-text or abstract? Examining topic coherence scores using latent Dirichlet allocation. In: 2017 IEEE International Conference on Data Science and Advanced Analytics (DSAA). IEEE; 2017.

[24] ANSA. Sospensione precauzionale del vaccino AstraZeneca anche in Italia; 2020. Available from: https://www.ansa.it/sito/notizie/cronaca/2021/03/15/astrazeneca-sequestrato-un-lotto-in-piemonte-e-uno-in-veneto_63bbe1c8-1e4e-4d20-b07f-a57b457b8bf7.html.

[25] giornale I. Cases of thrombosis. A storm: six countries already reject Astrazeneca; 2020. Available from: https://www.ilgiornale.it/news/mondo/gi-6-paesi-dicono-no-astrazeneca-ecco-cosa-sta-succedendo-1930332.html.

[26] STATISTA. Distribution of Twitter users worldwide as of April 2021, by age group; 2021. Statista. https://www.statista.com/statistics/283119/age-distribution-of-global-twitter-users/.

[27] Padilla JJ, Kavak H, Lynch CJ, Gore RJ, Diallo SY. Temporal and spatiotemporal investigation of tourist attraction visit sentiment on Twitter. PLoS One. 2018;13(6):e0198857. doi: 10.1371/journal.pone.0198857 29902270 PMC6002102

[28] Kwok SWH, Vadde SK, Wang G. Tweet topics and sentiments relating to COVID-19 vaccination among Australian Twitter users: Machine learning analysis. J Med Internet Res. 2021;23(5):e26953. doi: 10.2196/26953 33886492 PMC8136408

[29] Broniatowski DA, Jamison AM, Qi S, AlKulaib L, Chen T, Benton A, et al. Weaponized health communication: Twitter bots and Russian trolls amplify the vaccine debate. Am J Public Health. 2018;108(10):1378–1384. doi: 10.2105/AJPH.2018.304567 30138075 PMC6137759

[30] Gilani Z, Farahbakhsh R, Tyson G, Wang L, Crowcroft J. Of Bots and Humans (on Twitter). In: Proceedings of the 2017 IEEE/ACM International Conference on Advances in Social Networks Analysis and Mining 2017. ASONAM’17. New York, NY, USA: Association for Computing Machinery; 2017. p. 349–354. Available from: 10.1145/3110025.3110090.

[31] Scannell D, Desens L, Guadagno M, Tra Y, Acker E, Sheridan K, et al. COVID-19 vaccine discourse on Twitter: A content analysis of persuasion techniques, sentiment and mis/disinformation. J Health Commun. 2021;26(7):443–459. doi: 10.1080/10810730.2021.1955050 34346288

[32] Guess AM, Nyhan B, O’Keeffe Z, Reifler J. The sources and correlates of exposure to vaccine-related (mis)information online. Vaccine. 2020;38(49):7799–7805. doi: 10.1016/j.vaccine.2020.10.018 33164802 PMC7578671

[33] Piedrahita-Valdés H, Piedrahita-Castillo D, Bermejo-Higuera J, Guillem-Saiz P, Bermejo-Higuera JR, Guillem-Saiz J, et al. Vaccine hesitancy on social media: Sentiment analysis from June 2011 to April 2019. Vaccines (Basel). 2021;9(1):28. doi: 10.3390/vaccines9010028 33430428 PMC7827575

[34] Monselise M, Chang CH, Ferreira G, Yang R, Yang CC. Topics and sentiments of public concerns regarding COVID-19 vaccines: Social media trend analysis. J Med Internet Res. 2021;23(10):e30765. doi: 10.2196/30765 34581682 PMC8534488

[35] Fazel S, Zhang L, Javid B, Brikell I, Chang Z. Harnessing Twitter data to survey public attention and attitudes towards COVID-19 vaccines in the UK. Sci Rep. 2021;11(1):23402. doi: 10.1038/s41598-021-02710-4 34907201 PMC8671421

[36] Huangfu L, Mo Y, Zhang P, Zeng DD, He S. COVID-19 vaccine tweets after vaccine rollout: Sentiment-based topic modeling. J Med Internet Res. 2022;24(2):e31726. doi: 10.2196/31726 34783665 PMC8827037

